# Lithotomy Operations

**Published:** 1864-10

**Authors:** 


*Lithotomy Operations.*—Mr. Borlaise Childs has peformed his thirty-third consecutive case of lithotomy, followed by complete recovery and without the recurrence of any serious symptoms. The only peculiarities in Mr. Child's mode of operating, are, that he does not inject the bladder, but directs the patient to retain his urine for some time before the operation; and that he incises the bladder to a very small extent, just sufficient to admit of the fore-finger being used as a dilator.

